# Rosiglitazone Attenuated Endothelin-1-Induced Vasoconstriction of Pulmonary Arteries in the Rat Model of Pulmonary Arterial Hypertension via Differential Regulation of ET-1 Receptors

**DOI:** 10.1155/2014/374075

**Published:** 2014-02-18

**Authors:** Yahan Liu, Xiao Yu Tian, Yu Huang, Nanping Wang

**Affiliations:** ^1^Institute of Cardiovascular Science, Peking University Health Science Center, Beijing 100191, China; ^2^Institute of Vascular Medicine, Li Ka Shing Institute of Health Sciences, School of Biomedical Sciences, Chinese University of Hong Kong, Hong Kong; ^3^Cardiovascular Research Center, Xi'an Jiaotong University, Xi'an, Shaanxi 710061, China

## Abstract

Pulmonary arterial hypertension (PAH) is a fatal disease characterized by a progressive increase in pulmonary arterial pressure leading to right ventricular failure and death. Activation of the endothelin (ET)-1 system has been demonstrated in plasma and lung tissue of PAH patients as well as in animal models of PAH. Recently, peroxisome proliferator-activated receptor **γ** (PPAR**γ**) agonists have been shown to ameliorate PAH. The present study aimed to investigate the mechanism for the antivasoconstrictive effects of rosiglitazone in response to ET-1 in PAH. Sprague-Dawley rats were exposed to chronic hypoxia (10% oxygen) for 3 weeks. Pulmonary arteries from PAH rats showed an enhanced vasoconstriction in response to ET-1. Treatment with PPAR**γ** agonist rosiglitazone (20 mg/kg per day) with oral gavage for 3 days attenuated the vasocontractive effect of ET-1. The effect of rosiglitazone was lost in the presence of _L_-NAME, indicating a nitric oxide-dependent mechanism. Western blotting revealed that rosiglitazone increased ET_B_R but decreased ET_A_R level in pulmonary arteries from PAH rats. ET_B_R antagonist A192621 diminished the effect of rosiglitazone on ET-1-induced contraction. These results demonstrated that rosiglitazone attenuated ET-1-induced pulmonary vasoconstriction in PAH through differential regulation of the subtypes of ET-1 receptors and, thus, provided a new mechanism for the therapeutic use of PPAR**γ** agonists in PAH.

## 1. Introduction

Pulmonary arterial hypertension (PAH) is characterized by a progressive increase of pulmonary vascular resistance, leading to right ventricular failure and death [1]. ET-1 plasma level was elevated in the patients and experimental models for PAH [2, 3]. Expression of ET-1 was increased in lung tissues of PAH patients, predominantly in pulmonary arteries [4, 5]. ET-1 has 2 major subtypes of receptors: ET-A receptor (ET_A_R) is expressed on vascular smooth muscle cells (SMCs) and mediates vasoconstriction, whereas ET-B receptor (ET_B_R) is predominantly expressed in endothelial cells (ECs), where it primarily mediates vasodilatation and the clearance of ET-1. Expression of ET_A_R was upregulated in the lung tissues and pulmonary arteries from PAH patients with a well-established pathophysiological role [6–8]. However, a role of ET_B_R was rather controversial with the reports of unaltered, increased, or decreased expressions in the vessel tissues from various PAH conditions [9–15].

Emerging evidence suggests that peroxisome proliferator-activated receptor-*γ* (PPAR*γ*) agonists might have therapeutic role in treating PAH [16]. PPAR*γ* regulates the transcription of genes involved in glucose and lipid metabolism, inflammation, as well as vascular remodeling [17–19]. The expression of PPAR*γ* was reduced in the lungs from the PAH patients and the rat models [20, 21]. Similarly, mice with deletion of PPAR*γ* in SMCs or ECs developed PAH. Pharmacological activation of PPAR*γ* ameliorated PAH. [21–25]. In ECs, PPAR*γ* activators inhibited thrombin- or oxidized low-density lipoproteins- (LDL-) induced ET-1 production [26, 27]. In particular, we recently observed that PPAR*γ* agonist rosiglitazone attenuated ET-1-induced vasoconstriction through upregulation of ET_B_R in ECs [28]. However, whether the regulation of ET_B_R accounts for the ameliorative effect of PPAR*γ* agonists in PAH arteries remains to be elucidated. In the present study, we examined the role of rosiglitazone on ET-1-induced vasocontraction of pulmonary arteries in rat PAH models and the underlying mechanism.

## 2. Materials and Methods

### 2.1. Animals, Cell Culture, and Reagents

Male Sprague-Dawley rats were used and the experiments were conducted in accordance with the National Institutes of Health (NIH) Guide for the Care and Use of Laboratory Animals with the approval by the institutional committee. Polyclonal rabbit anti-ET_B_R antibody was from Abcam. Polyclonal rabbit anti-ET_A_R was from Santa Cruz Biotechnology. ET-1 and *N* ^G^-nitro-_L_-arginine methyl ester (_L_-NAME) were from Sigma-Aldrich Co., rosiglitazone was from GlaxoSmithKline, and A192621 was from Abbott Laboratories.

### 2.2. Chronic Hypoxia Induced PAH in Rat

Rats were exposed to normobaric hypoxia (10% oxygen) or normoxia (21% oxygen) for 3 weeks and then treated with rosiglitazone (20 mg/kg per day) or water with oral gavage for 3 days.

### 2.3. Isometric Tension Measurement

Left lungs were removed and placed in oxygenated Krebs-Henseleit solution. Pulmonary arteries were carefully dissected from adjacent connective tissue and cut into several ring segments of ≈2 mm long for measuring isometric force. Organ chambers (Multi Myograph System, Danish Myo Technology A/S) were filled with (37°C) Krebs solution containing (in mmol/L) 119.0 NaCl, 4.7 KCl, 2.5 CaCl_2_, 1.0 MgCl_2_, 25.0 NaHCO_3_, 1.2 KH_2_PO_4_, and 11.0 _D_-glucose. The Krebs solution in the organ bath was initially open to room air, being bubbled with mixed 95% O_2_ and 5% CO_2_. Each ring was suspended between 2 tungsten wires (diameter, 40 *μ*m) in the chamber under optimal resting tension (2.5 mN as previously determined for the pulmonary arteries) and left for 90-minute equilibration. Vasoreactivity was measured to compare contractions in response to ET-1 (1 to 50 nmol/L) in the absence and presence of _L_-NAME (100 *μ*mol/L). The effects of antagonist of ET_B_R were tested on ET-1-induced contractions.

### 2.4. Western Blot Analysis

Pulmonary arteries were dissected, frozen in liquid nitrogen, and homogenized in RIPA lysis buffer containing protease inhibitors. Protein lysates separated on 12.5% sodium dodecyl sulfate polyacrylamide gels (SDS-PAGE) and transferred to PVDF membranes, which were blocked with 5% nonfat milk in Tris-buffered saline-Tween (0.2%) (TBS-T) for 1 h, incubated overnight with primary antibody and then horseradish peroxidase-(HRP-) conjugated secondary antibody, and visualized with ECL reagent.

### 2.5. Statistical Analysis

Results represent mean ± SEM. Comparisons among groups involved ANOVA followed by unpaired Student's *t*-test. *P* < 0.05 was considered statistically significant.

## 3. Results

### 3.1. Rosiglitazone Ameliorated ET-1-Mediated Vasoconstriction in Rats with PAH

**Figure 1:**
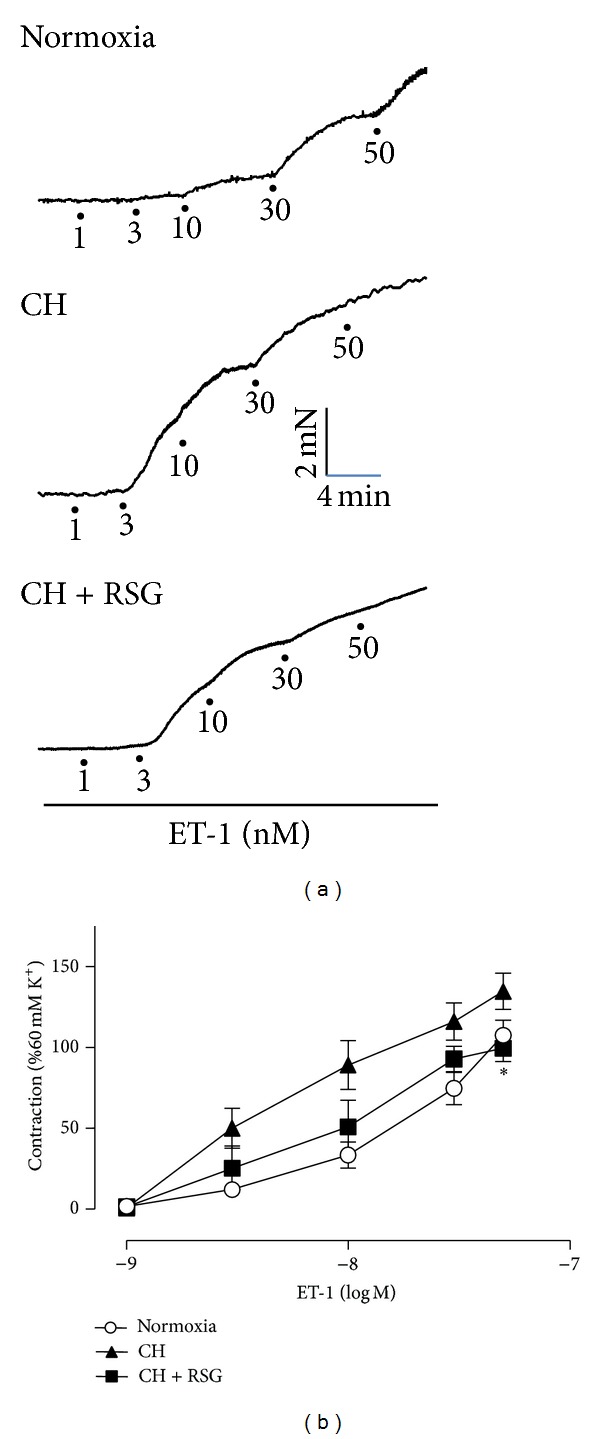
(a) Representative recordings of ET-1-induced contractions of pulmonary arteries from normoxia-, chronically hypoxic- (CH-), or rosiglitazone- (RSG-) treated CH-rats. (b) RSG ameliorated ET-1-mediated vasoconstriction in pulmonary arteries from the rats with PAH. Data were mean ± SEM from 5 to 7 rats. *P < 0.05 CH + RSG versus CH group.

**Figure 2:**
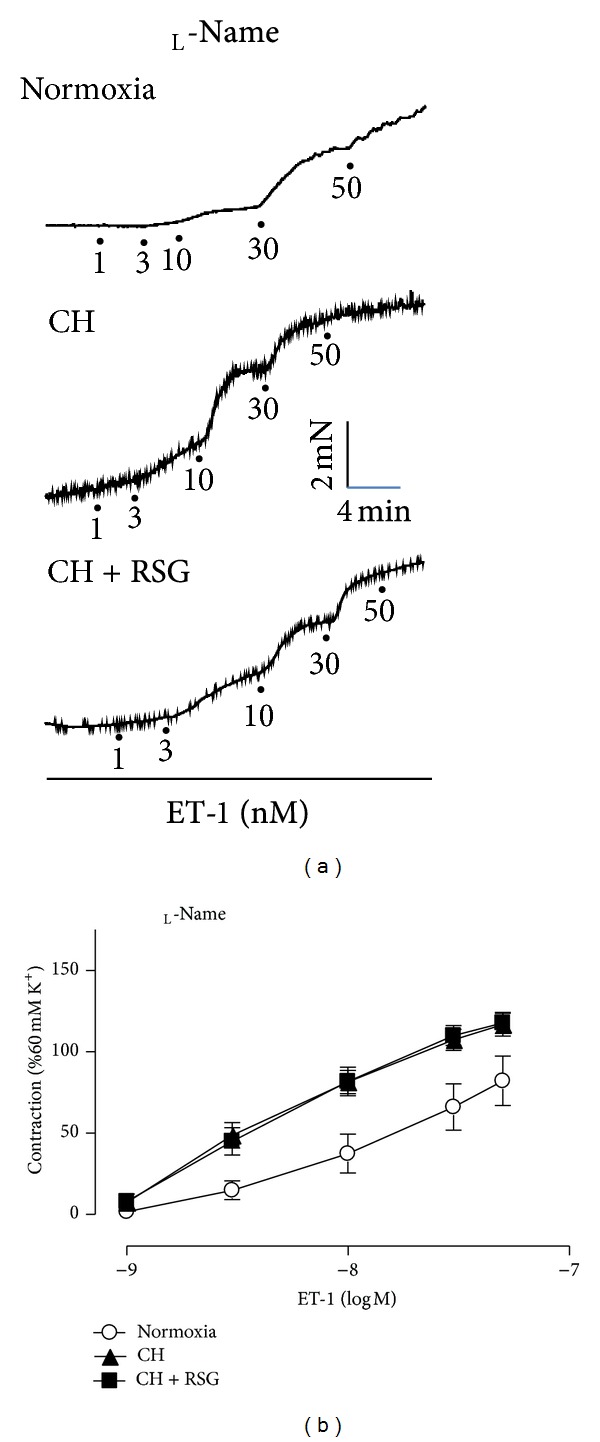
(a) Representative recordings of ET-1-induced contractions pretreated with _L_-NAME (100 *μ*mol/L) in pulmonary arteries from normoxia-, CH-, or RSG-treated CH-rats. (b) The effect of RSG on ET-1-mediated vasoconstriction was abrogated in the presence of _L_-NAME (100 *μ*mol/L). Data were mean ± SEM from 5 to 7 rats.

To investigate the effect of rosiglitazone on vasoconstriction of pulmonary arteries induced by ET-1, pulmonary arteries from normoxia-, chronic hypoxia- (CH-), and rosiglitazone-treated CH-rats were dissected from groups of animals for isometric tension measurement responding to ET-1. The ET-1-induced contractions in pulmonary arteries were elevated in PAH rats compared to the normoxic rats. Treatment with PPAR*γ* agonist rosiglitazone (20 mg/kg per day) reversed the vasocontractive effect of ET-1 (Figure 1). However, this effect of rosiglitazone was abolished by the treatment with the inhibitor of endothelial nitric oxide synthase (eNOS) _L_-NAME, indicating a NO-dependent mechanism (Figure 2).

### 3.2. Rosiglitazone Increased ET_B_R Protein Levels in Pulmonary Arteries from PAH Rats

**Figure 3:**
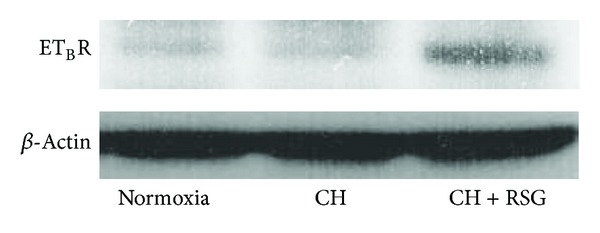
Rosiglitazone upregulated ET_B_R expression in rats with PAH. Western blotting was performed with the protein samples extracted from the pulmonary arteries of normoxia-, CH-, or RSG-treated CH-rats. Data shown are representative of three independent experiments.

To understand the mechanism for the effect of rosiglitazone on ET-1-induced vasocontraction in pulmonary arteries, we examined the protein level of ET_B_R with Western blotting. As shown in Figure 3, ET_B_R protein level was unaltered in the pulmonary arteries from CH-induced PAH rats. However, rosiglitazone treatment increased the expression of ET_B_R. In contrast, it reduced the expression of ET_A_R (Supplemental Figure 2 available online at http://dx.doi.org/10.1155/2014/374075).

### 3.3. Inhibitory Effect of Rosiglitazone Is Abolished by ET_B_R Antagonist

**Figure 4:**
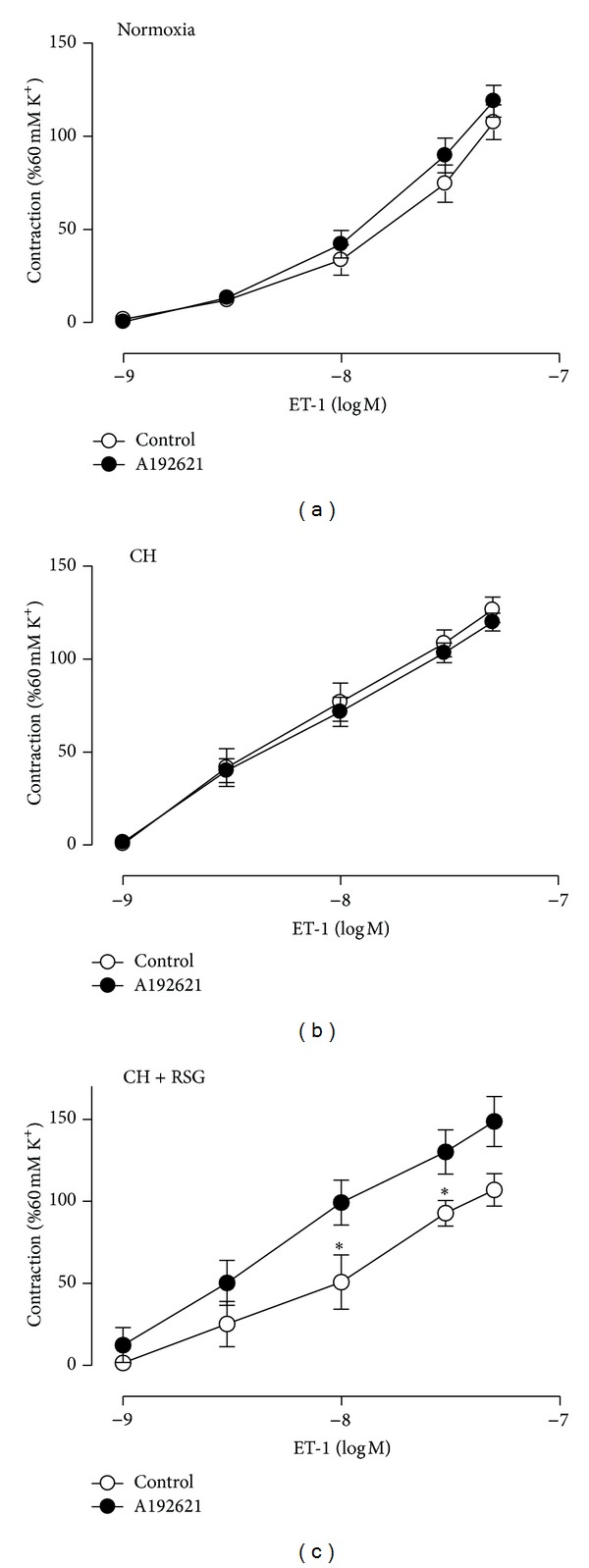
The ameliorative effect of RSG on ET-1-induced contractions was abrogated by ET_B_R antagonist. Concentration-dependent contractions to ET-1 pretreated with ET_B_R antagonist A192621 (10 nmol/L) in pulmonary arteries from normoxia- (a), CH (b), or RSG-treated CH (c) rats. Data were mean ± SEM from 5 to 8 rats. *P < 0.05 versus control.

To examine the functional role of ET_B_R in mediating the rosiglitazone effect on ET-1-induced vasoconstriction, pulmonary arteries were dissected from normoxia-, CH-, and rosiglitazone-treated CH-rats to measure the ET-1-responsive isometric tension in the presence or absence of A192621, a selective ET_B_R antagonist. In normoxic and PAH rats, A192621 (10 nmol/L) did not significantly alter the ET-1-induced contraction (Figures 4(a) and 4(b)). However, in the rosiglitazone-treated pulmonary arteries, A192621 abolished the ameliorative effect on the ET-1-induced vasocontraction (Figure 4(c)).

## 4. Discussion

The vascular effects of ET-1 are mediated by 2 pharmacologically distinct G protein-coupled receptors, ET_A_R and ET_B_R [29]. ET_A_R is mostly expressed in SMCs and mediates the vasoconstrictive and proliferative effects of ET-1 [30]. However, ET_B_R expressed in ECs mediates endothelial-dependent vasodilatation by stimulating the production of NO and prostacyclin, prevents apoptosis, and promotes the clearance of ET-1 [31, 32]. ET_B_R is present in low densities on vascular smooth muscle cells where its activation induces vasoconstriction [33, 34]. Since ET_B_R elicits vasodilation and vasoconstriction, its vascular functions in pulmonary arterial hypertension need to be further characterized. ET_B_R-deficient rats developed exacerbated PAH after exposure to chronic hypoxia, characterized by elevated pulmonary arterial pressure, diminished cardiac output, increased right ventricular hypertrophy, and increased total pulmonary resistance. Plasma ET-1 level and mRNA of ET-converting enzyme-1 (ECE-1) were much higher in lungs from ET_B_R-deficient rats compared with control rats. In ET_B_R-deficient rats, the pulmonary vessels showed less endothelial NO synthase (eNOS) and NO production, supporting a role of NO in ET_B_R-mediated vasodilation in the pulmonary vasculature [35]. Other studies in monocrotaline (MCT) induced PAH rats also showed that ET_B_R deficiency accelerated the progression of PAH and neointimal lesion [36, 37]. Although both ET_A_R antagonist (ambrisentan) and dual ET_A_R/ET_B_R antagonist (bosentan) have been approved for treatment of PAH [38], selective antagonists for ET_A_R and ET_B_R appeared to have different effects on PAH. In a dog model for PAH, ET_B_R antagonist RES-701-1 was found to increase pulmonary arterial pressure whereas sarafotoxin S6c, an ET_B_R agonist, decreased pulmonary arterial resistance [39]. In addition, ET_B_R antagonist also elevated ET-1 concentrations in both in vivo and in vitro studies [40]. These findings suggest that activation of ET_B_R may play a protective role in the PAH.

In addition to three categories of FDA-approved treatments including prostanoids, ET-1 receptor antagonists, and phosphodiesterase 5 (PDE5) inhibitors, PPAR*γ* agonists thiazolidinediones (TZDs) including rosiglitazone and pioglitazone have shown beneficial effects in animal models of PAH. In rodent PAH models induced by MCT or hypoxia and those associated with insulin resistance, TZDs were found to effectively reduce pulmonary arterial pressure and right ventricular hypertrophy [21, 22, 24, 25, 41]. Recently, we showed that rosiglitazone reversed pulmonary arterial remodeling and inhibited vasoconstriction in response to serotonin in the rat PAH models induced by MCT and hypoxia. Although the molecular mechanisms underlying the TZD effects on PAH development remain unclear, a generally accepted hypothesis is that TZDs may act via their receptor PPAR*γ* to modulate the expression of key genes involved in the pathogenesis of PAH such as ET-1, eNOS, p27^KIP1^, adiponectin, apoE, MMP, and RhoA/ROCK. In this study, we provided in vivo evidence that rosiglitazone ameliorated ET-1-induced vasocontraction in the pulmonary arteries of PAH rats (Figure 1). The ameliorative effect of rosiglitazone was mediated via differential regulation of ET-1 receptors. In particular, the upregulation of ET_B_R might play a major role because rosiglitazone treatment increased the expression of ET_B_R in the pulmonary arteries (Figure 3) and A192621, a selective antagonist of ET_B_R, abrogated the effect (Figure 4). Conversely, rosiglitazone inhibited the induction of ET_A_R in the pulmonary arteries of PAH rats (Supplemental Figure 2). It is conceivable that rosiglitazone may have the vasoprotective effects by altering the ratio of ET_A/B_ receptors. ET_B_R in ECs may increase Ca^2+^ influx and the activation of eNOS, which leads to the production of NO and induction of vascular relaxation. This notion is corroborated with the result that the effect of rosiglitazone was abolished in the presence of _L_-NAME, an inhibitor of eNOS (Figure 2). Importantly, the induction of endothelial ET_B_R is considered to be a PPAR*γ*-specific mechanism as we previously identified ET_B_R to be a direct target gene of PPAR*γ* [28].

## 5. Conclusions

In conclusion, we demonstrated that rosiglitazone upregulated the expression of ET_B_R, which mediated the decreased vasoconstriction in the rat models of PAH. This finding suggested a new mechanism for the protective role of PPAR*γ* in the development of PAH.

## Supplementary Material

Figure 1.Vascular remodeling in rats for pulmonary arterial hypertension (PAH). Weigert's elastic staining revealed medial thickening changes in lungs. CH, chronically hypoxic.
Figure 2 Rosiglitazone inhibited ETAR expression in rats for PAH. Western blot analyses of protein levels of ET_A_R in rat pulmonary arteries. CH, chronically hypoxic. RSG, rosiglitazone.
