# Supplementary material for: Rosiglitazone Attenuated Endothelin-1-Induced Vasoconstriction of Pulmonary Arteries in the Rat Model of Pulmonary Arterial Hypertension via Differential Regulation of ET-1 Receptors
Source: PPAR Res. 2014 Feb 18;2014:374075. doi: 10.1155/2014/374075 (PMC3950948; doi:10.1155/2014/374075)
Supplement: Supplementary file 1 — Figure 1.Vascular remodeling in rats for pulmonary arterial hypertension (PAH). Weigert's elastic staining revealed medial thickening changes in lungs. CH, chronically hypoxic. Figure 2 Rosiglitazone inhibited ETAR expression in rats for PAH. Western blot analyses of protein levels of ETAR in rat pulmonary arteries. CH, chronically hypoxic. RSG, rosiglitazone. [file 374075.f1.pptx]

## Slide 1
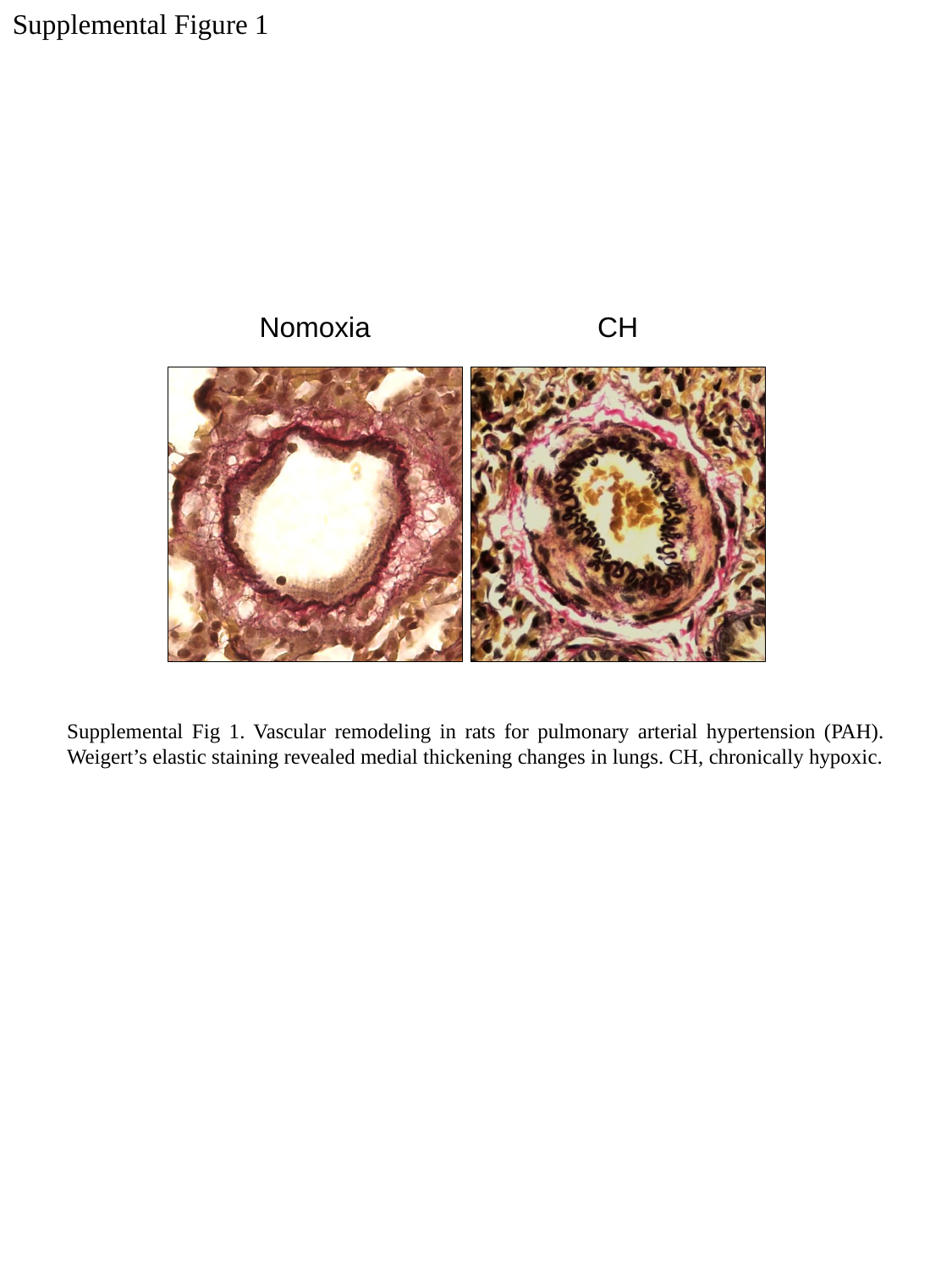

Supplemental Figure 1
Nomoxia
CH
Supplemental Fig 1. Vascular remodeling in rats for pulmonary arterial hypertension (PAH). Weigert’s elastic staining revealed medial thickening changes in lungs. CH, chronically hypoxic.

## Slide 2
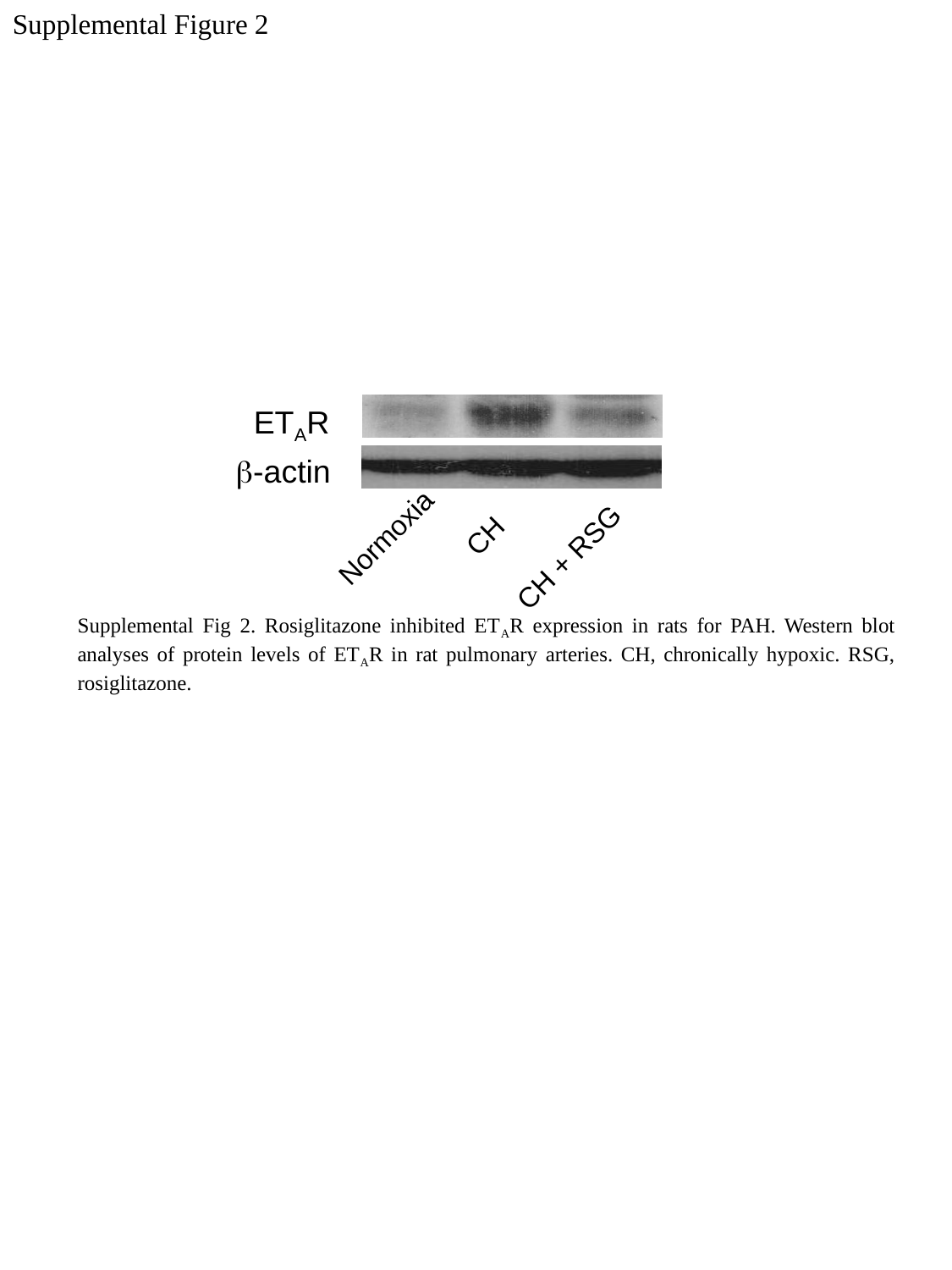

Supplemental Figure 2
ETAR
b-actin
CH
Normoxia
CH + RSG
Supplemental Fig 2. Rosiglitazone inhibited ETAR expression in rats for PAH. Western blot analyses of protein levels of ETAR in rat pulmonary arteries. CH, chronically hypoxic. RSG, rosiglitazone.
